# Improving the Corrosion Resistance of Wrought ZM21 Magnesium Alloys by Plasma Electrolytic Oxidation and Powder Coating

**DOI:** 10.3390/ma14092268

**Published:** 2021-04-27

**Authors:** Lavanya Rani Ballam, Hamed Arab, Massimiliano Bestetti, Silvia Franz, Giulia Masi, Ramona Sola, Lorenzo Donati, Carla Martini

**Affiliations:** 1 Department of Chemistry, Materials and Chemical Engineering “Giulio Natta”, Politecnico di Milano, 20133 Milan, Italy; lavanyarani.ballam@mail.polimi.it (L.R.B.); arab.hamed@polimi.it (H.A.); massimiliano.bestetti@polimi.it (M.B.); silvia.franz@polimi.it (S.F.); 2 Department of Civil, Chemical, Environmental and Materials Engineering, University of Bologna, 40131 Bologna, Italy; giulia.masi5@unibo.it; 3 Department of Industrial Engineering, University of Bologna, 40136 Bologna, Italy; ramona.sola@unibo.it (R.S.); l.donati@unibo.it (L.D.)

**Keywords:** micro arc oxidation, anodizing, spark anodization, salt spray, corrosion resistance, fluoride free electrolyte

## Abstract

Plasma Electrolytic Oxidation (PEO) was applied to extruded ZM21 Mg alloys to improve their corrosion resistance in a chloride-containing environment. PEO was carried out in DC mode and voltage control in a fluoride-free electrolyte. Potentiodynamic polarization tests in 3.5 wt.% NaCl aqueous solution and neutral salt spray (NSS) tests were carried out. Microstructural and profilometric characterization, as well as NSS tests were performed in different conditions: (i) bare ZM21, (ii) PEO-treated ZM21, (iii) powder-coated ZM21 (without PEO interlayer), and (iv) PEO-treated ZM21 with powder coating top layer (carboxyl-functionalized polyester resin). The PEO + powder coating double layer was identified as the best-performing corrosion protection.

## 1. Introduction

High strength-to-weight ratio, high damping capacity, and good castability make Mg alloys attractive for lightweighting in automotive and aerospace [1,2]. Mg-Zn alloys are among the most promising Mg alloys, with the main advantage of high formability, combined with moderate strength and corrosion resistance [3]. Among Mg-Zn alloys, ZM21 is a promising candidate for automotive applications due to its extrusion speeds being higher than other medium strength alloys such as AZ31B [4], thus allowing for a cost reduction in the production of Mg extrusions and coping with one of the strategic objectives of the automotive industry [5]. Unfortunately, localized corrosion induced by non-homogeneous distribution of second-phase particles and impurities may significantly affect the ZM21 alloy in aggressive environments [6] and requires the use of surface modifications to achieve satisfactory service life and industrial application.

The corrosion protection of Mg alloys can be obtained by a wide range of surface modification methods, such as thermal spray, electrochemical, and electroless plating, sol-gel, chemical, and physical vapor deposition coatings, surface alloying, chemical conversion, and anodizing [7,8]. Among anodizing treatments, Plasma Electrolytic Oxidation (PEO), alternatively referred to as Micro-Arc Oxidation (MAO) or Anodic Spark Deposition (ASD), has been successfully applied to Mg alloys. PEO is a high-voltage process, carried out above the dielectric breakdown potential of the anodic oxide, where controlled micro-arc discharges assist and facilitate the growth of an adherent oxide layer, which may incorporate components of the electrolyte, leading to improved corrosion and wear resistance [9,10,11,12,13,14,15]. For these reasons, as well as for its cost-effectiveness and relatively low environmental impact, PEO is widely applied in the automotive and aerospace sector. A few recent papers reported on the successful application of PEO to ZM21 alloy [15,16,17,18]. Only lab-scale processes were described, where the samples had a simple geometry like flat coupons [15,16], plates [17] or cylinders [18] and the use of fluoride-based compounds in the electrolyte was mostly required [16,18]. Moreover, investigations on the corrosion resistance were limited to electrochemical tests such as polarization and/or electrochemical impedance spectroscopy (EIS), either in simulated body fluid [16,18] or in aqueous NaCl solution [15,17]. All these studies indicate that the porosity and intrinsic defectivity of the PEO layers detrimentally affect corrosion resistance. In order to move closer to the industrial application of ZM21 alloys, in the present work a PEO process carried out in a fluoride-free electrolyte, has been applied on flat coupons and then to real components in the form of extruded profiles. The corrosion behavior was assessed not only by potentiodynamic polarization tests in 3.5 wt.% NaCl aqueous solution but also by neutral salt spray tests. Even though the mechanical and tribological properties of the anodic layers obtained by PEO on Mg alloys are improved by incorporation of electrolyte species and densification due to microdischarges, the overall structure of PEO layers remains rather porous. Therefore, sealing the anodic layer is still required for adequate corrosion behavior in aggressive environments [10]. For this reason, also the influence of a commercial powder coating top layer as a further corrosion protection on the PEO-treated surface was investigated.

## 2. Materials and Methods

### 2.1. Characterization of the ZM21 Alloys

Commercial ZM21 alloy was provided by SHL-Alubin Ltd. (Kiryat Bialik, Israel) in the form of extruded profiles with square section (30 mm × 30 mm, length: 170 mm) and a wall thickness of 2 mm. The profiles were hot-extruded (billet temperature: 416 °C, maximum temperature of mandrel and bearings: 450 and 330 °C, respectively) with a speed of 3.5 m/min. These conditions provided an average grain size lower than 20 μm. Flat samples (30 mm × 30 mm, henceforward “sacrificial samples” for instrumental characterization purposes), were extracted from extruded profiles for the first step of the work, i.e., the optimization of PEO treatment conditions.

The chemical composition of ZM21 was determined by Glow Discharge Optical Emission Spectroscopy (GDOES) using a Spectruma GDA 650 analyzer (Spectruma Analytik GmbH, Hof, Germany) with a Grimm-style glow discharge lamp in DC mode, operating at 900 V and 9 mA in an argon atmosphere at 3 hPa. The internal diameter of the tubular anode (hence the analyzed area in each measurement) was 4.0 mm. Table 1 reports compositional data, averaged over three analyzed sites. No deviations from nominal composition data [19] were observed.

The microstructural characterization of the as-supplied ZM21 alloy was carried out on metallographic samples prepared according to the ASTM E3 standard [20] and chemically etched with Nital 2 (100 mL ethanol and 2 mL nitric acid) for a few seconds, according to the ASTM E407 standard [21]. Microstructural analyses were carried out by means of a Zeiss (Oberkochen, Germany) Axio Imager light optical microscope (LOM) and a Zeiss (Carl Zeiss Jena GmbH, Jena, Germany) EVO50 scanning electron microscope (SEM), equipped with an Oxford Instrument (Abingdon, UK) INCA X-Act PentaFet Precision energy dispersive spectroscope (EDS). Image analysis was carried out by the ^®^Image Pro-Plus software (version 4.5) to evaluate the average grain size of the as-supplied alloy according to ASTM E 112 [22], as well as to determine the thickness of PEO and powder coating layers.

### 2.2. Coating of the ZM21 Alloys

PEO was carried out in an aqueous solution containing 100g/L (0.26 M) sodium phosphate tribasic dodecahydrate (Na_3_PO_4_∙12H_2_O), 20 g/L (0.16 M) sodium metasilicate (Na_2_SiO_3_), and 25 g/L (0.06 M) sodium tetraborate decahydrate (Na_2_B_4_O_7_∙10H_2_O). The treatment was carried out in a Pyrex^®^ cell, the total volume of the electrolyte being 1 L. The anodic exposed area was 9900 mm^2^. Two AISI 316 steel plates with an exposed area of 18,000 mm^2^ were used as cathodes. Before PEO the ZM21 specimens were cleaned with acetone and rinsed in distilled water. PEO was carried out ramping the potential at a constant scan rate of 1 V/s from 0 to 170 V by means of a DC power supply (Essedikappa, Milano, Italy). At visual observation, during the treatment gas evolution and sparking occurred over the whole surface of the anode. The total processing time was 188 s. During PEO, the electrolyte temperature was kept at 30 °C and controlled by means of a cryostat (FP50-HL, JULABO Italia Srl, Milano, Italy). ZM21 specimen having an exposed area of 1500 mm^2^ were also anodized for 600 s for instrumental characterization purposes (“sacrificial samples”).

The PEO treatment was subsequently applied to extruded profiles. A commercial thermosetting polymeric powder coating (carboxyl-functionalized polyester resin) was deposited on the PEO-treated surface as a further corrosion protection top layer, a widely established procedure for industrial applications of Mg alloys [23].

### 2.3. Characterization of the Coated ZM21 Alloys

After PEO, the surface morphology of the sacrificial specimens and of the extruded profiles was investigated by scanning electron microscopy (SEM, EVO 50, Carl Zeiss Jena GmbH, Jena, Germany) and the elemental composition was assessed by energy dispersive X-ray spectroscopy (EDS). The thickness of the coating on sacrificial samples was measured exploiting the eddy current principle by means of a Dual scope FMP100 probe (Helmut Fischer Gmbh, Institut Für Elektronik Und Messtechnik, Sindelfingen, Germany). X-ray diffraction (XRD) patterns were acquired using a PW1830 instrument (Malvern Panalytical Ltd., Malvern, UK and Almelo, Netherlands) operating in Bragg–Brentano geometry at a potential of 40 kV with a filament current of 40 mA. Spectra were acquired in the 2θ range 20–90° at the scanning rate of 2.5° min^−1^ using the Cu Kα_1_ radiation. The XRD patterns were indexed according to the powder diffraction files released by the International Centre for Diffraction Data (U.S.A.) for Magnesium (PDF 35-0821), Periclase (MgO) (PDF 87-0653) and Quartz (SiO_2_) (PDF 01-0649) phases. To evaluate the corrosion resistance of the PEO coatings, potentiodynamic (PD) polarization tests were carried out both on the bare and PEO-treated specimen. PD tests were performed in 3.5 wt.% NaCl aqueous solutions at a scan rate of 1 mV s^−1^ and 25 °C. Before potentiodynamic tests, the open circuit potential of the specimen was monitored for 600 s. A conventional three-electrode cell was used, where the anodized specimen was the working electrode, a platinum sheet was the counter electrode, and a saturated calomel electrode (SCE) (Amel S.r.l., Milano, Italy) was the reference electrode. All the measurements were performed using an Amel mod. 2549 potentiostat/galvanostat (Amel S.r.l., Milano, Italy).

In the extruded profiles, the thickness of PEO layers and powder coatings was measured by image analysis of unetched polished cross-sections (according to the procedure for porous coatings described in [24]) and mean values were calculated by at least 5 measurements at the same magnification. The phase composition of the oxidized layers was determined by X-ray diffraction (XRD), performing θ–2θ scans from 10° to 100° with a 0.02 step size and a 4 s dwell time, by a PANalytical Expert PRO X-ray diffractometer (Malvern Panalytical Ltd., Malvern, UK and Almelo, Netherlands) with X’Celerator detector (Philips Analytical, Almelo, Netherlands) and a Ni-filtered Cu-Kα radiation source (λ = 0.15405 nm), operated at 40 kV and 40 mA.

Topographic analysis was carried out on the free surface of the extruded profiles by stylus profilometry (Hommelwerke T2000, Schwenningen, Germany; tip radius: 5 μm), in order to characterize surface topography.

Scratch tests were carried out on the anodic oxides formed on extruded ZM21 profiles, using a Revetest Xpress Plus device (CSM Instruments, Peseux, Switzerland) equipped with a Rockwell diamond indenter (spherical tip radius: 200 μm). Progressive load scratch tests were carried out from 1 to 30 N, with a linear speed rate of 10 mm/min and a scratch length of 10 mm. Scratch morphology was observed by light microscopy during scratching and by 3-D digital microscopy (Hirox KH 7700, Tokyo, Japan) and SEM after scratching.

A neutral salt spray (NSS) test was carried out in accordance with the international standard ISO 9227:2006 [25], using a 5 wt.% NaCl solution and a total time of exposure of 120 h (DCTC 600P Angelantoni, Massa Martana (PG), Italy). Before testing, all the untreated surfaces of exposed samples were coated with a polyimide film (Kapton), producing one-surface samples for each of the four conditions: (1) bare ZM21 (reference), (2) PEO-treated ZM21, (3) powder-coated ZM21, (4) PEO-treated ZM21 with powder coating top layer. Samples were inclined of 20° from the vertical to avoid accumulation of the salt solution during exposure. A visual inspection of the samples was performed during and at the end of the exposure. Moreover, at the end of NSS test light and SEM/EDS microscopy was performed on the corroded surfaces and on polished cross sections of corroded samples.

## 3. Results and Discussion

### 3.1. Microstructure of ZM21

**Figure 1:**
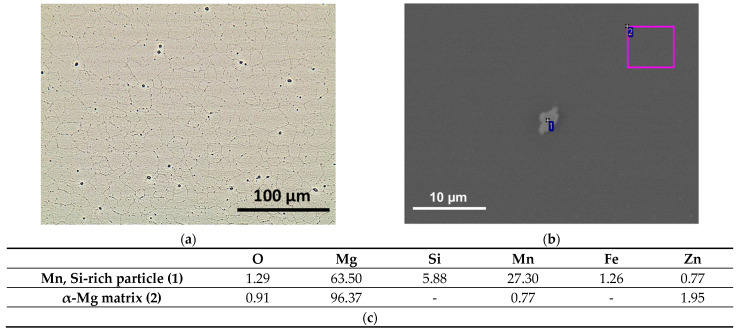
Microstructure of the ZM21 substrate: (**a**) optical image and (**b**) higher magnification SEM image with (**c**) EDS analysis (wt.%) of the unetched alloy.

Optical and SEM images showing the microstructure of the ZM21 alloy are shown in Figure 1a,b: a fully recrystallized microstructure was observed in the plane perpendicular to the extrusion direction. Equiaxed grains displayed an average size of 9 ± 5 μm and a bimodal grain size distribution was observed, consisting of both relatively coarse grains (15 ± 4 μm) and fine grains (3 ± 1 μm), as already reported by other authors for ZM21 after extrusion [26]. Relatively small Mn,Si-rich particles (<5 μm, as shown by high magnification SEM image in Figure 1b, accompanied by localized EDS analysis results, Figure 1c) were also detected in the α-Mg matrix (with Zn and Mn in solid solution, as indicated by EDS data in Figure 1c). Mn,Si-rich particles also contained Fe, a common impurity in Mg alloys. Similar observations were reported for the same alloy in the extruded condition, also by Bian et al. [6]. The formation of the Zn-rich β phase was not observed, due to the balance between Zn and Mn in the alloy [27].

### 3.2. PEO Treatment of ZM21

**Figure 2:**
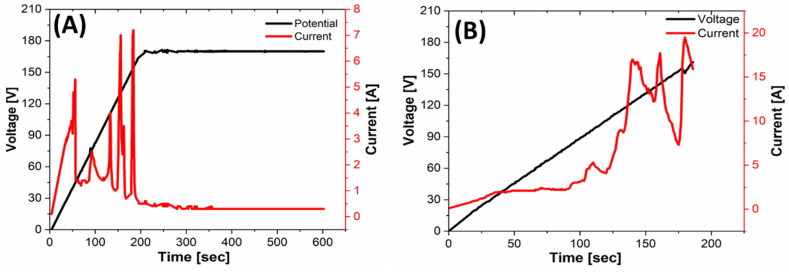
Potential (black) and current (red) curves as a function of the PEO processing time for the ZM21 sacrificial specimen (**A**) and for the ZM21 extruded profile (**B**).

The chronopotentiometric and chronoamperometric curves recorded during the PEO of the sacrificial samples (Figure 2A) showed a typical current profile characterized by randomly distributed peaks corresponding to discharge avalanches on the anodic surface. The current peaks did not exceed 7 A during the PEO of the sacrificial samples (Figure 2A). This induced lower energy dissipation and a better temperature control. During PEO of the extruded profiles (Figure 2B), the current peaked at 20 A, probably because of the higher anodic surface area. Correspondingly, a more intense sparking and gas evolution was observed, with significant electrolyte temperature increase, inducing the operator to interrupt the treatment at the beginning of the potential plateau. The discrepancy on the chronoamperometric profiles reported in Figure 2A,B can be ascribed to the different ratio of anodic to cathodic exposed area. This issue would deserve a dedicated development stage, which was out of the scope of this work.

**Figure 3:**
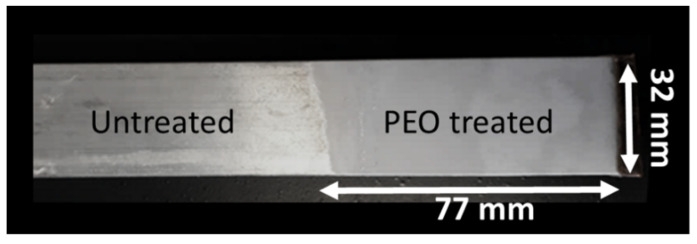
Representative image of a PEO-treated ZM21 extruded profile.

As shown in Figure 3, at visual examination the oxide film formed on the surface of the anodized ZM21 extruded profile appeared homogeneous.

**Figure 4:**
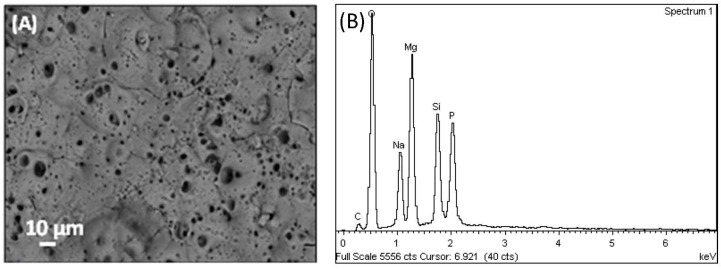
PEO-treated surface: (**A**) SEM image and (**B**) EDS surface spectrum of the oxide layer obtained onto a ZM21 sacrificial sample at 170 V, 188 s, 30 °C in 100g/L Na_3_PO_4_∙12H_2_O), 20 g/L Na_2_SiO_3_, 25 g/L Na_2_B_4_O_7_∙10H_2_O.

After anodizing, the surface of sacrificial samples exhibited a micrometric and sub-micrometric porosity; however, the typical cracks often resulting from PEO of Mg alloys [16] were not observed (Figure 4A). The EDS analysis of the surface (Figure 4B) revealed that the PEO layer contained O, Zn, Na, Mg, Si and P, with Zn and Mg from the ZM21 alloy and Na, Si and P incorporated from the electrolyte.

**Figure 5:**
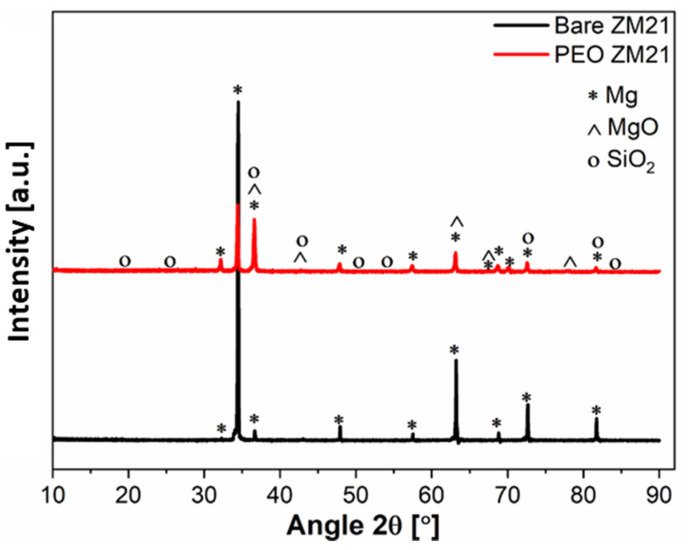
XRD pattern of the bare ZM21 alloy (black) and of the anodic film (red) obtained onto ZM21 at 170 V, 188 s, 30 °C in 100g/L Na_3_PO_4_∙12H_2_O), 20 g/L Na_2_SiO_3_, 25 g/L Na_2_B_4_O_7_∙10H_2_O.

XRD patterns shown in Figure 5 demonstrate that the PEO film consisted of a mixture of MgO and SiO_2_.

### 3.3. Microstructural and Micromechanical Charaterization of Extruded Profiles

**Figure 6:**
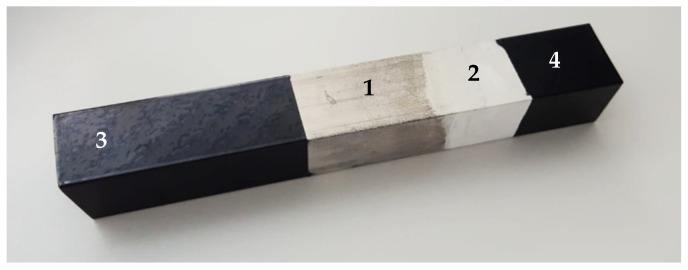
General view of extruded profiles (30 × 30 mm^2^, length: 170 mm), with indication of the investigated conditions: (1) bare ZM21, (2) PEO-treated ZM21, (3) powder-coated ZM21, (4) PEO-treated ZM21 with powder coating top layer.

A general view of extruded profiles, with indication of the investigated conditions is shown in Figure 6: (1) bare ZM21 (reference), (2) PEO-treated ZM21, (3) powder-coated ZM21 (without PEO interlayer), (4) PEO-treated ZM21 with powder coating top layer.

Surface roughness parameters (R_a_, R_q_, R_z_, R_t_ and R_max_ according to [28], measured in the areas highlighted in Figure 6) are reported in Table 2.

The profilometric data in Table 2 show that PEO induced a typical R_a_ increase (about 5 times) by comparison to the untreated substrate, due to the peculiar morphology of the anodic oxide discussed below. The deposition of the powder coating decreased the roughness of both the bare alloy and the PEO-treated surface, due to the levelling power of the thick polymeric layer.

**Figure 7:**
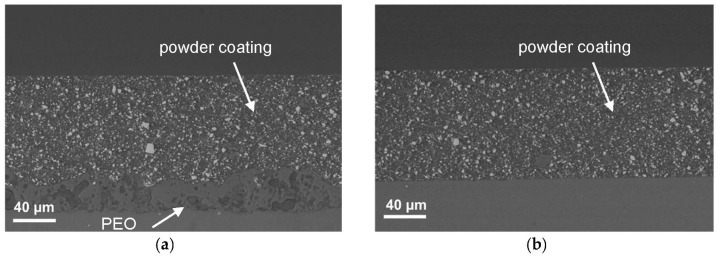
Cross-section BSE images of (**a**) PEO-treated and powder-coated ZM21 and (**b**) powder-coated ZM21 (without PEO interlayer).

**Figure 8:**
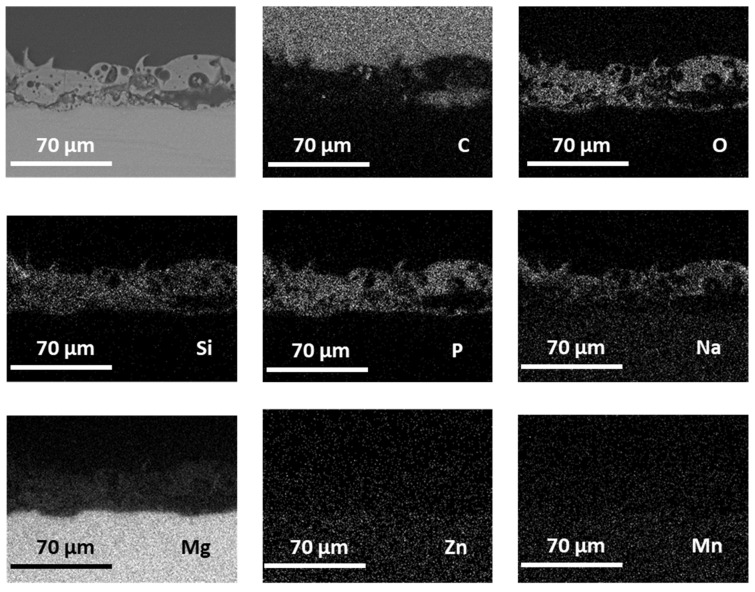
BSE image (top left) and X-ray EDS elemental maps of the PEO-treated ZM21 extruded profile (polished cross section; the presence of C is due to the mounting resin).

The surface morphology of PEO-treated profile and the corresponding cross-section image are shown in Figure 4A, Figure 7 and Figure 8, respectively. The surface morphology appeared rough and porous with typical craters generated by local discharge events occurring during the growth of the anodic oxide layer [15,29]. A few microcracks, derived from thermal stresses, and a pancake morphology, formed by molten oxide erupted from discharge channel and rapidly cooled by the electrolyte at the room temperature, were also observed on the surface. As the coating grows in thickness, the pores that already exist on the surface can be filled by oxides generated in the subsequent micro arc discharges.

The cross-section images (Figure 7 and Figure 8) showed that the PEO layer/substrate interface is slightly undulated, due to local thickening of the anodic oxide following breakdown events [11]. The pores are unevenly distributed throughout the specimen cross-section and the average thickness of the film is about 30 μm. Typical discharge channels, pores and cracks, bigger and larger in the outermost region, were also observed. The characteristic anodic oxide morphology afforded mechanical interlocking of the powder coating top layer (Figure 7a), an important prerequisite for satisfactory bonding strength. Additionally, the deposition of the powder coating top layer allowed to obtain a smoother surface (as shown also by surface roughness data in Table 2), levelling out the rough PEO surface. The average thickness of the PEO layer was 39 ± 4 µm, while the powder coating top layer was 118 ± 9 µm thick (Figure 7a,b), regardless the presence of the PEO interlayer (as expected, since the powder coating was applied in a single painting session). The coating thickness was fairly uniform in both cases (PEO layer alone and PEO with powder coating top layer). EDS analysis of bright spots in the BSE cross-section images of the powder coating showed the presence of Ba-and Ca-based compounds, typical inorganic fillers added to the powder coating top layer (such as BaSO_4_ and CaCO_3_).

Elemental X-ray maps recorded by EDS on polished cross sections of PEO-treated samples (Figure 8) showed a homogeneous distribution of alloy elements (Mg, Zn, Mn) as well as of elements incorporated from the electrolyte (O, Si, P).

The small Mn,Si-rich particles described in Section 3.1 (Figure 1b) do not appear to have any significant influence on the overall growth of the PEO layer at this scale, and the ZM21 alloy basically responds to oxidation as a single phase material. As for the elements from the electrolyte, elemental maps show that Na-rich species are mostly located in the outer areas of the coating, while Si and P are more homogeneously distributed throughout the whole layer. The distribution of these elements in PEO layers is influenced by ionic mobility of different species, as discussed by Monfort et al. and Matykina et al. in [30,31].

**Figure 9:**
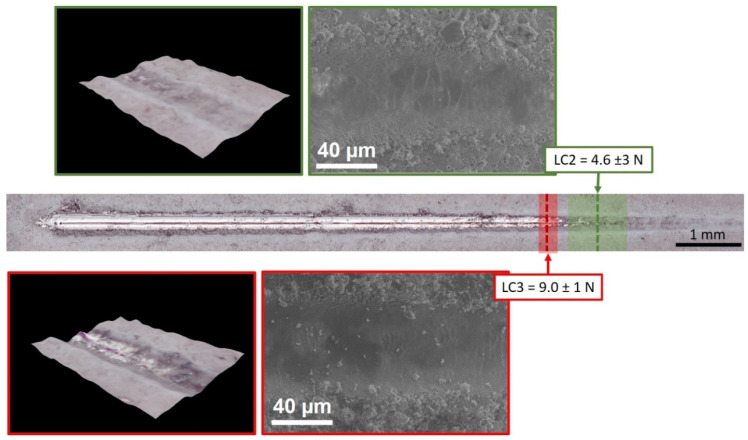
Overview of a representative scratch track on PEO-treated ZM21 extruded profiles (optical image), with indication of critical loads Lc2 (first coating detachment) and Lc3 (full delamination). Each critical load for adhesive failure of the PEO layer is accompanied by the corresponding SEM image (SE) and 3D-digital microscopy image of the scratch track (black background).

Representative images of scratch tracks with indication of critical loads Lc2 and Lc3 for adhesive failure of PEO layer, estimated according to ISO 20502:2016 [32] are reported in Figure 9.

The measured average value of Lc2, indicating when the coating detachment begins, and Lc3, corresponding to penetration of the coating to the substrate at the center of the track, were 4.6 ± 3 N and 9.0 ± 1 N respectively, comparable to those measured in a previous work on PEO-treated EV31A Mg alloy, where scratching was carried out in the same conditions [29]. Substrate and coating properties influence the failure mode during scratch testing [33]: PEO-treated Mg alloys can be considered as hard coating on soft substrate even though the hardness difference may not be notably high. Therefore, PEO layers were bent into the track as a consequence of substrate plastic deformation. The SEM image corresponding to Lc2 in Figure 9 (top right) shows buckling failures (cracks and patches of coating detachment) appearing in the scratch track, in the areas where the indenter movement generates pile-up deformation. After full delamination of the PEO layer, only substrate plastic deformation was observed, together with scattered micro-fragments from the anodic oxide (bottom right SEM image in Figure 9).

### 3.4. Electrochemical Corrosion Tests on PEO-Treated ZM21

**Figure 10:**
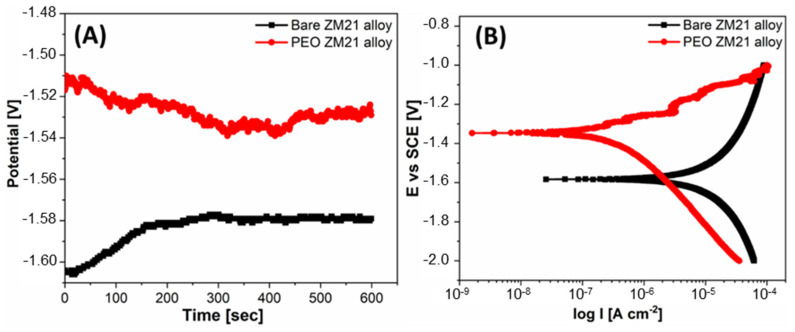
Open circuit voltage as a function of immersion time (**A**) and potentiodynamic curves (**B**) of ZM21 before (black curve) and after (red curve) PEO. Tests carried out in 3.5% NaCl aqueous solutions.

Corrosion tests were carried out on sacrificial specimens. Open circuit measurements in 3.5% NaCl solutions (Figure 10A) showed that the immersion potential of the anodized sample is more positive than that of the bare sample. The potentiodynamic polarization data calculated by means of the Tafel fit using EC-Lab software are reported in Table 3, together with the polarization resistance (*R_p_*) calculated using the Stern–Geary equation [16]:

$$R_{p}=\beta_{a}\times \beta_{c}/2.303i_{corr}\left(\beta_{a}+\beta_{b}\right)$$

According to the potentiodynamic tests (Figure 10B), after anodization the corrosion current density decreased by one order of magnitude, i.e., from 2.5∙10^−8^ A cm^−2^ to 1.5∙10^−9^ A cm^−2^ and the corrosion potential increased by about 300 mV, i.e., from −1.6 V vs. SCE (untreated ZM21 alloy) to −1.3 V vs. SCE (anodized alloy). Correspondingly, the polarization resistance of the alloy increased by two orders of magnitude after the PEO treatment, rising from about 9837 Ω cm^2^ to about 145,848 Ω cm^2^.

### 3.5. NSS Test on Extruded Profiles

**Figure 11:**
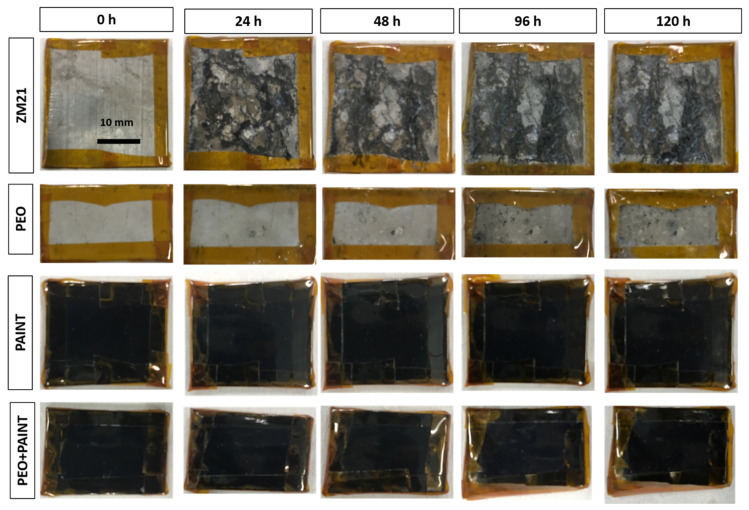
Visual observation of the samples before and during the NSS test.

**Figure 12:**
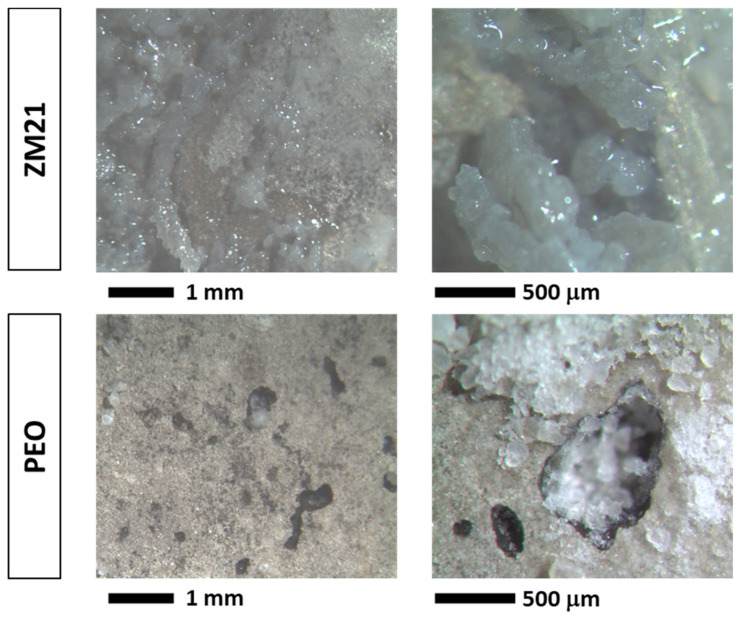
Macroscopic images of corroded surfaces (bare ZM21 and PEO-treated ZM21) at the end of the NSS test (120 h).

The results of the visual inspection of the samples during and at the end of NSS test are reported in Figure 11. Already after a few hours of exposure (>6 h), ZM21 alloy exhibited corrosion products on the surfaces, indicating the high reactivity of this material in NaCl solution. At the end of the exposure, the reference ZM21 samples showed a strongly damaged surfaces characterized by the precipitation of uniformly distributed corrosion products, as highlighted in micrographs of Figure 12. The PEO-treated samples showed localized corrosion attacks for short exposure time (>6 h) that are clearly visible after 24 h. However, these corrosion attacks on PEO surfaces did not significantly evolve with time. This is probably due to the initiation of the localized corrosion attacks in correspondence with PEO treatment defects (e.g., porosity). In addition, corrosion products continuously precipitated inside the localized corrosion sites, mitigating the corrosion rate of the process. However, at a longer exposure time (>96 h), a localized acceleration of the corrosion process was observed with a consequently leaching of the corrosion products, as shown in Figure 12. Conversely, for both the painted samples, no corrosion attacks and/or painting damages were observed within the 120 h NSS test, indicating an increased performance for both the painted samples. However, longer NSS test exposure will be necessary for discerning the performance of the painted samples on the ZM21 alloy or on the PEO-treated surfaces.

**Figure 13:**
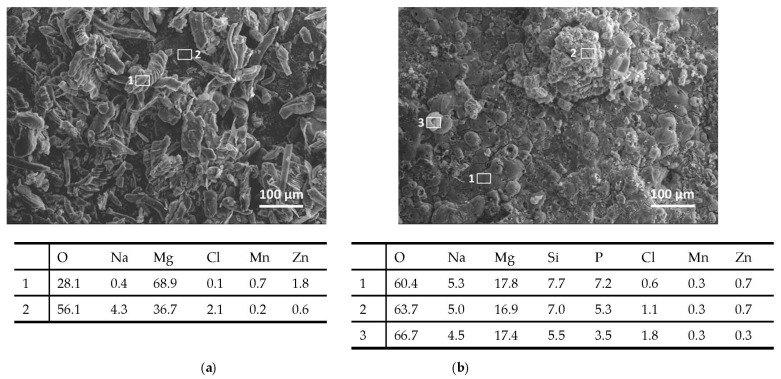
SEM images (secondary electrons) and localized EDS analyses (wt.%) of corroded surfaces (bare ZM21 (**a**) and PEO-treated ZM21 (**b**)) at the end of the NSS test (120 h).

The corroded surface as well as the polished cross section of the bare and PEO-treated ZM21 were also analyzed by SEM/EDS at the end of the NSS test (120 h). Figure 13 shows the morphology of corroded surfaces and further demonstrates that the PEO layer contributed to limit the formation of corrosion products on ZM21: a more extensive formation of Cl- and O-rich crystalline corrosion products was observed on bare ZM21 (Figure 13a) than on the PEO-treated surface (Figure 13b). However, some localized corrosion attacks are also visible on the PEO-treated surface (areas 2 and 3 in Figure 13b), with increased amounts of elements from the aggressive environment (namely Cl and O) by comparison to the less corroded areas of the anodic oxide (area 1 in Figure 13b), which still retains its typical surface morphology with volcano-like features (as shown before corrosion by Figure 4A). 

**Figure 14:**
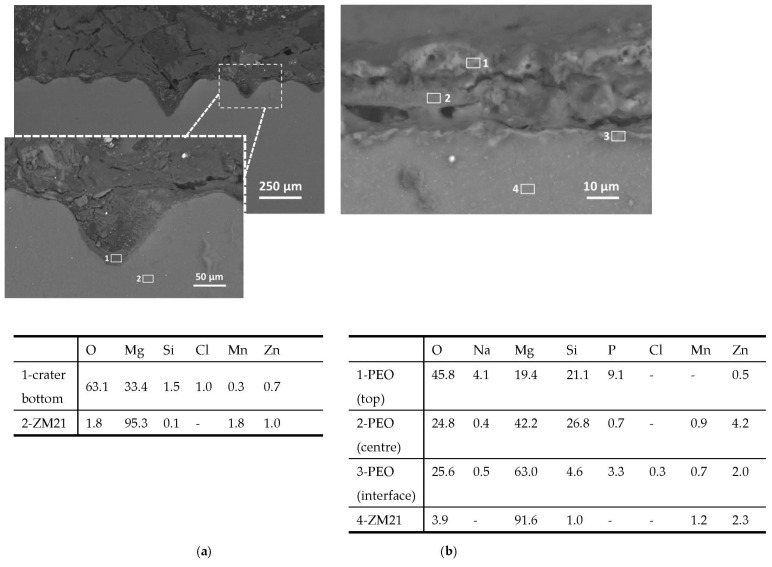
SEM images (backscattered electrons) and localized EDS analyses (wt.%) of polished cross sections through corroded samples (bare ZM21, with general view and high magnification detail (**a**) and PEO-treated ZM21, high magnification detail (**b**)) at the end of the NSS test (120 h).

Figure 14 compares the polished cross section of the bare and PEO-treated ZM21, showing that while the bare alloy underwent remarkable localized corrosion, with the formation of several craters (Figure 14a), the PEO-treated samples still showed the presence of the anodic oxide layer, even though with a slight increase of defects (cracks and pores), which favored the previously mentioned localized corrosion phenomena. Cl from the aggressive environment was always detected at the interface between the corrosion products and the alloy, as expected. 

## 4. Conclusions

PEO coatings were produced on ZM21 extruded profiles using a fluoride-free electrolyte and were investigated in terms of microstructure, phase composition, scratch and corrosion resistance. In addition, a polymeric topcoat (thermosetting carboxyl-functionalized polyester resin) was deposited by powder coating onto PEO layers. The following conclusions can be drawn from this work:

- Without the polymeric topcoat, the PEO layer, consisting of a mixture of MgO and SiO_2_ and showing a satisfactory adhesion to the substrate, was able to reduce corrosion current by one order of magnitude in 3.5 wt.% NaCl aqueous solution, increasing the polarization resistance of the alloy by two orders of magnitude.
- During neutral salt spray tests, even though the PEO-treated samples showed localized corrosion attacks at short exposure time, starting in correspondence with PEO layer defects, the precipitation of corrosion products inside the corrosion sites mitigated the process. Without the polymeric top layer, however, at longer exposure time localized acceleration of the corrosion process was observed. The deposition of the polymeric top layer, well-adhering on the rough PEO interlayer and acting as an effective barrier coating, afforded the best corrosion performance in salt spray environment.

Based on the obtained results, the PEO + powder coating combination proved to be the most promising for the improvement of corrosion resistance of the ZM21 extruded alloy in chloride-containing environment. This was due to the ability of the thick powder coating to seal all the defects in the PEO layer, which in turn afforded improved adhesion of the polymeric top layer.

## Figures and Tables

**Table 1:** Chemical composition (wt.%) of the ZM21 magnesium alloy.

Mg	Zn	Mn	Other
97.15 ± 0.05	1.59 ± 0.04	1.12 ± 0.02	0.14

**Table 2:** Surface roughness parameters (μm) measured in different areas (see Figure 7) of the ZM21 extruded profile.

	R_a_	R_q_	R_z_	R_t_	R_max_
Bare ZM21 (1)	0.9 ± 0.3	1.4 ± 0.6	6.7 ± 2.5	11.1 ± 3.1	10.3 ± 3.5
PEO-treated ZM21 (2)	4.8 ± 0.2	6.1 ± 0.3	28.2 ± 0.6	34.4 ± 1.8	31.8 ± 1.1
Powder-coated ZM21 (3)	0.4 ± 0.1	0.5 ± 0.1	1.0 ± 0.1	2.0 ± 0.1	1.5 ± 0.1
PEO-treated ZM21 + powder coating (4)	2.7 ± 0.5	3.3 ± 0.2	13.8 ± 1.5	21.3 ± 0.8	19.8 ± 0.9

**Table 3:** Open circuit potential (E_ocp_), corrosion potential (E_corr_), corrosion current density (i_corr_), anodic (*β_a_*) and cathodic (*β_c_*) and resistance polarization (*R_p_*) of ZM21 alloys before and after PEO.

Sample	E_ocp_ (mV)	E_corr_ (mV)	i_corr (_µA/cm^2^)	*β*_*a*_ (mV)	*β*_*c*_ (mV)	*R_p_* (Ω Cm^2^)
Bare ZM21 alloy	−1583.7	−1581.7	25	298.4	298.8	9837.47
PEO-treated ZM21 alloy	−1527.7	−1339.1	1.5	116.8	240.3	145,848.2

## Data Availability

The data presented in this study are available on request from the corresponding author. The data are not publicly available due to confidential reasons.

## References

[1] Friedrich H., Schumann S. (2001). Research for a “new age of magnesium” in the automotive industry. J. Mater. Process. Technol..

[2] Pantelakis S.G., Alexopoulos N.D., Chamos A.N. (2007). Mechanical performance evaluation of cast magnesium alloys for automotive and aeronautical applications. J. Eng. Mater. Technol..

[3] Maeng D.Y., Kim T.S., Lee J.H., Hong S.J., Seo S.K., Chun B.S. (2000). Microstructure and strength of rapidly solidified and extruded Mg-Zn alloys. Scr. Mater..

[4] Housh S., Mikucki B., Stevenson A. (1990). Selection and Application of Magnesium and Magnesium Alloys. ASM Handbook, Volume 2: Properties and Selection: Nonferrous Alloys and Special-Purpose Materials.

[5] Osborne R., Powell B. Magnesium Vision 2020: A North American Automotive Strategic Vision for Magnesium. https://www.tms.org/Communities/FTAttachments/MG_2020_-_Released_11_1_[1].1.06.pdf.

[6] Bian D., Jiang J., Zhou W., Li N., Zheng Y., Leeflang S., Zhou J. (2018). In vitro characterization of ZM21 mini-tube used for biodegradable metallic stent. Mater. Lett..

[7] Gray J.E., Luan B. (2002). Protective coatings on magnesium and its alloys-A critical review. J. Alloys Compd..

[8] Shaw B.A., Wolfe R.C., Crame S.D., Covino B.S. (2005). Corrosion of Magnesium and Magnesium-Base Alloys. ASM Handbook, Volume 13B: Corrosion: Materials.

[9] Barati Darband G., Aliofkhazraei M., Hamghalam P., Valizade N. (2017). Plasma electrolytic oxidation of magnesium and its alloys: Mechanism, properties and applications. J. Magnes. Alloy..

[10] Blawert C., Bala Srinivasan P., Dong H. (2010). Plasma electrolytic oxidation treatment of magnesium alloys. Surface Engineering of Light Alloys.

[11] Arrabal R., Matykina E., Hashimoto T., Skeldon P., Thompson G.E. (2009). Characterization of AC PEO coatings on magnesium alloys. Surf. Coatings Technol..

[12] Franz S., Arab H., Lucotti A., Castiglioni C., Morini F., Bestetti M. (2020). Exploiting direct current plasma electrolytic oxidation to boost photoelectrocatalysis. Catalysts.

[13] Da Forno A., Bestetti M. (2010). Effect of the electrolytic solution composition on the performance of micro-arc anodic oxidation films formed on AM60B magnesium alloy. Surf. Coatings Technol..

[14] Da Forno A., Bestetti M., Lecis N. (2013). Effect of anodising electrolyte on performance of AZ31 and AM60 magnesium alloys microarc anodic oxides. Trans. Inst. Met. Finish..

[15] Hari Krishna K., Koteswara Rao S.R., Dondapati S., Rameshbabu N. (2019). Influence of Plasma Electrolytic Oxidation on Corrosion Characteristics of Friction Stir Welded ZM21 Magnesium Alloy. Prot. Met. Phys. Chem. Surfaces.

[16] Sreekanth D., Rameshbabu N., Venkateswarlu K., Subrahmanyam C., Rama Krishna L., Prasad Rao K. (2013). Effect of K2TiF6 and Na2B4O7 as electrolyte additives on pore morphology and corrosion properties of plasma electrolytic oxidation coatings on ZM21 magnesium alloy. Surf. Coatings Technol..

[17] Ram Kumar V., Muthupandi V. (2019). Effect of Electrolyte in Microarc Oxidation on Providing Corrosion Resistance to Inhomogeneous Microstructure in ZM21 Magnesium Alloy. Trans. Indian Inst. Met..

[18] Kwiatkowski L., Kapuścińska A., Bałkowiec A., Lutze R. (2015). Increasing the surface functionality of Mg alloys by means of plasma electrolytic oxidation. Solid State Phenom..

[19] Standardization Administration of China (2016). Designation and composition of wrought Magnesium and Magnesium Alloys.

[20] ASTM (2017). Standard Guide for Preparation of Metallographic Specimens.

[21] ASTM (2015). Standard Practice for Microetching Metals and Alloys.

[22] ASTM (2013). Standard Test Methods for Determining Average Grain Size.

[23] Morri A., Ceschini L., Martini C., Bernardi A. (2020). Influence of plasma electrolytic oxidation on fatigue behaviour of ZK60A-T5 magnesium alloy. Coatings.

[24] Tonelli L., Martini C., Ceschini L. (2017). Improvement of wear resistance of components for hydraulic actuators: Dry sliding tests for coating selection and bench tests for final assessment. Tribol. Int..

[25] International Standard (2017). Corrosion Tests in Artificial Atmospheres. Salt Spray Tests.

[26] Mostaed E., Fabrizi A., Dellasega D., Bonollo F., Vedani M. (2015). Microstructure, mechanical behavior and low temperature superplasticity of ECAP processed ZM21 Mg alloy. J. Alloys Compd..

[27] Yang Z., Li J.P., Zhang J.X., Lorimer G.W., Robson J. (2008). Review on Research and Development of Magnesium Alloys. Acta Metall. Sin. English Lett..

[28] International Standard (1997). Geometrical Product Specifications (GPS)-Surface Texture: Profile Method-Rules and Procedures for the Assessment of Surface Texture.

[29] Tonelli L., Pezzato L., Dolcet P., Dabalà M., Martini C. (2018). Effects of graphite nano-particle additions on dry sliding behaviour of plasma-electrolytic-oxidation-treated EV31A magnesium alloy against steel in air. Wear.

[30] Monfort F., Matykina E., Berkani A., Skeldon P., Thompson G.E., Habazaki H., Shimizu K. (2007). Species separation during coating growth on aluminium by spark anodizing. Surf. Coatings Technol..

[31] Matykina E., Arrabal R., Scurr D.J., Baron A., Skeldon P., Thompson G.E. (2010). Investigation of the mechanism of plasma electrolytic oxidation of aluminium using 18O tracer. Corros. Sci..

[32] International Standard (2016). Fine Ceramics (Advanced Ceramics, Advanced Technical Ceramics). Determination of Adhesion of Ceramic Coatings by Scratch Testing.

[33] Bull S.J., G-Berasetegui E. (2006). An overview of the potential of quantitative coating adhesion measurement by scratch testing. Tribol. Int..

